# BEDROOM ENVIRONMENTAL QUALITY PARAMETERS ASSOCIATED WITH OLDER ADULTS’ BEHAVIORAL AND PSYCHOLOGICAL SYMPTOMS

**DOI:** 10.1093/geroni/igae098.4129

**Published:** 2024-12-31

**Authors:** Wan-Tai Au-Yeung, Zachary Beattie, Jeffrey Kaye

**Affiliations:** Oregon Health and Science University, Portland, Oregon, United States; Oregon Health & Science University, Portland, Oregon, United States; Oregon Health & Science University, Portland, Oregon, United States

## Abstract

Behavioral and psychological symptoms are common among older adults with apathy, depression and anxiety being the most prevalent. Identifying factors associated with such symptoms may help mitigate them. The study, Monitoring Apathy, Depression and Anxiety Using Technology Evaluations, aimed to identify indoor environmental quality factors that may trigger these symptoms. The study was a 9-month observational study in 12 participants’ personal residences. Participant mean age was 79.0 (6.4) years, 7 were males (58.3%), 7 had normal cognition, 4 had MCI and 1 had early-stage AD. The participants self-reported the severity of their apathy, depression and anxiety weekly online by answering standard surveys sent via email, which consisted of the 6-item Withdrawal-Apathy-Vigor subscale from the Geriatric Depression Scale for assessing apathy, and depression, and anxiety subscales from Patient-Reported Outcomes Measurement Information System for assessing depression and anxiety respectively. Bedroom environments were assessed with environmental sensors (Awair Omni, San Francisco) which measured light level, noise level, temperature, humidity and air quality every 5 minutes. Individual mixed-effect models were built with each symptom severity being the outcome, and each weekly mean bedroom environmental quality factor being the predictor, while controlling for age, sex and the week the data was collected. Lower light level in the bedroom was significantly associated with more severe depression scores (p< 0.01) and higher temperature was associated with more severe apathy scores (p< 0.05). Optimizing specific aspects of indoor environmental quality in older adults’ personal homes can potentially assist in improving management of behavioral and psychological symptoms.

